# Redox-Dependent Effects in the Physiopathological Role of Bile Acids

**DOI:** 10.1155/2021/4847941

**Published:** 2021-09-04

**Authors:** Josué Orozco-Aguilar, Felipe Simon, Claudio Cabello-Verrugio

**Affiliations:** ^1^Laboratory of Muscle Pathology, Fragility, and Aging, Department of Biological Sciences, Faculty of Life Sciences, Universidad Andres Bello, Santiago 8370146, Chile; ^2^Millennium Institute on Immunology and Immunotherapy, Santiago 8370146, Chile; ^3^Center for the Development of Nanoscience and Nanotechnology (CEDENNA), Universidad de Santiago de Chile, Santiago 8350709, Chile; ^4^Millennium Nucleus of Ion Channel-Associated Diseases (MiNICAD), Universidad de Chile, Santiago 8370146, Chile; ^5^Laboratory of Integrative Physiopathology, Department of Biological Sciences, Faculty of Life Sciences, Universidad Andres Bello, Santiago 8370146, Chile

## Abstract

Bile acids (BA) are recognized by their role in nutrient absorption. However, there is growing evidence that BA also have endocrine and metabolic functions. Besides, the steroidal-derived structure gives BA a toxic potential over the biological membrane. Thus, cholestatic disorders, characterized by elevated BA on the liver and serum, are a significant cause of liver transplant and extrahepatic complications, such as skeletal muscle, central nervous system (CNS), heart, and placenta. Further, the BA have an essential role in cellular damage, mediating processes such as membrane disruption, mitochondrial dysfunction, and the generation of reactive oxygen species (ROS) and oxidative stress. The purpose of this review is to describe the BA and their role on hepatic and extrahepatic complications in cholestatic diseases, focusing on the association between BA and the generation of oxidative stress that mediates tissue damage.

## 1. Introduction

Bile acids (BA) are a group of steroidal molecules derived from cholesterol. These molecules have been historically described as solubilizing agents for lipids and activators for pancreatic enzymes, supporting their role in intestinal absorption [1, 2]. While the BA are intrinsically toxic in elevated concentrations due to the amphipathic structure, several antecedents indicate that BA also have endocrine and metabolic functions. Furthermore, despite their steroidal nature, the BA stereochemistry differs from other steroids, such as steroidal hormones. Therefore the receptor and signaling vary [3, 4].

Cholestatic liver diseases and the complications derived from the gradual destruction of bile ducts produce BA accumulation in the liver. This increment of BA induces a proinflammatory response and an increased production of reactive oxygen species (ROS), leading to cellular damage. Cholestatic pathologies do not have effective treatments, making them one of the leading causes of liver transplants [5–7].

Several pathological conditions, endogenous or xenobiotic-induced, might generate the obstruction of bile flow, elevating the BA concentrations within hepatocytes and serum and damaging the neighboring tissues [6, 8, 9]. In this line, the BA-dependent cytotoxicity and cellular alterations are associated with oxidative stress, mainly affecting the liver and extrahepatic tissues such as the heart, skeletal muscle, and placenta. In the central nervous system (CNS), contradictory effects of BA and their receptors reportedly show generation or prevention of oxidative stress [10–12].

This review presents a description of BA and their primary receptors, their clinical association with cholestatic diseases, and the impact of BA-induced oxidative stress observed in critical tissues.

## 2. Bile Acids

BA are amphiphilic molecules that belong to the acidic sterol family. They have a unique stereochemistry, hydroxyl groups, and an aliphatic side chain with a terminal carboxyl residue. All hydroxyl groups and the side carboxyl group are faced in the same plane, except in ursodeoxycholic acid (UDCA) (Table 1), forming a structure with opposing lipophilic properties [13]. BA correspond to the bile's significant lipidic component and are synthesized from cholesterol in the liver and secreted to store in the gallbladder [1, 13]. *De novo* synthesized BA, such as cholic acid (CA) and chenodeoxycholic acid (CDCA), are categorized as primary and are the most abundant species in humans. The primary BA can be conjugated with glycine or taurine at the side chain, increasing the water solubility before secretion into the canalicular duct. After the release into the small intestine, primary BA can be dehydroxylated by the intestinal microbiota, converting CA and CDCA into the secondary BA deoxycholic acid (DCA) and lithocholic acid (LCA), respectively. Also, the 7-hydroxyl group in CDCA can be epimerized to form the UDCA [4, 13–15].

The BA form micelles (in concentrations between 1 and 20 *μ*M) with hydrophobic compounds, facilitating absorption processes at the intestine. Besides, lipid absorption is favored by BA-dependent pancreatic lipase activation [14, 16]. Then, unconjugated and conjugated BA are reabsorbed in the small intestine and colon via passive and active transport back to the liver, completing the enterohepatic circulation [13, 16].

Further, BA have endocrine/metabolic functions, regulating their synthesis, transport, and detoxification; mediating the cellular energetics and lipid and glucose homeostasis; and modulating the intestinal microbiota [7, 13, 14, 16, 17].

Alterations in BA metabolism and transport lead to pathological conditions. For example, high levels of BA in enterohepatic circulation can damage the liver and intestine, generating jaundice, cholesterol gallstones, and cholestatic liver diseases. Conversely, BA deficiency leads to nutrient malabsorption and fat-soluble vitamin deficiency [1, 7, 13, 18]. Both extreme situations highlight the importance of a balanced BA metabolism due to their significant role in corporal homeostasis.

## 3. Bile Acid Receptors

The amphipathic nature of BA has been used to describe their significant physiological properties. However, the metabolic role of bile acids has been described mainly by discovering diverse receptors [1].

The BA receptors can be classified into two major groups: the nuclear and G-protein-coupled receptors. Below, we focused on the most widely described receptors, the farnesoid X receptor (FXR) and Takeda-G-protein-receptor-5 (TGR5) receptor, mentioning other receptors with a lesser expression and minor characterization in the literature (Table 2).

### 3.1. Farnesoid X Receptor

Initially, the FXR was recognized as a receptor for farnesol and some related metabolites. It forms a heterodimeric complex with the retinoid X receptor. In 1999, it was reported that BA are the physiologic ligands of FXR by three independent groups. The ligand-receptor interaction is independent of the conjugation status of BA, whereas the affinity of this interaction is determined by the substitutions in carbon 7 of BA [19–21]. FXR is encoded by the *fxr* gene that generates four transcripts' variants, all responsive to BA [22]. Another gene in mammals, *fxrβ* (pseudogene in humans and primates), expresses the FXR*β* receptor that senses mainly lanosterol, and to a minor extent, BA [23].

FXR is involved in the metabolism and regulation of BA levels. Thus, FRX diminishes BA synthesis by repressing the critical enzyme expression associated with this process, such as cytochrome (CYP) 7a1 and 12-*α*-hydroxylase [3, 24–26]. Also, FXR decreased intracellular levels of BA in hepatocytes by two mechanisms: downregulating the uptake transporters (SLCA1 and SLCO1A2) and upregulating the levels of efflux transporters (BSEP, MRP2, and OST*α*) [27–30]. Furthermore, FXR diminishes the intestinal absorption of BA by inhibiting the expression of apical sodium-dependent BA transporter, an uptake transporter from enterocytes in the ileum, colon, and jejunum [31].

FXR is also related to a protective effect in several tissues. For example, the absence of FXR expression has been associated with vacuolization and hepatocyte hypertrophy, and also with increased serum triglyceride, cholesterol, glucose, and BA (resulting in mild cholestasis) [3, 32–34]. Also, the absence of FXR expression affected cardiac function and elevated the levels of myocardial injury markers associated with a BA overload [35]. Similarly, in a diabetes mice model, the FXR knock-out aggravates cardiac fibrosis and lipid accumulation [36]. Furthermore, FXR agonists diminish cardiac fibrosis, kidney damage, and pancreatic hypertrophy and reduce lipid serum levels in obese/diabetic mice models, decreasing hepatic fibrosis and portal pressure in a nonalcoholic steatohepatitis rat model [37–39]. These antecedents demonstrate the importance of FXR on corporal function via homeostasis of BA, carbohydrates, and lipids.

### 3.2. TGR5

The primary membrane receptor for BA is the G-protein-coupled TGR5, also called BG37, GPBAR1, or M-BAR. There is a correlation between BA's hydrophobicity and affinity for TGR5. Besides, the TGR5 activity associates with elevated intracellular calcium levels and cytosolic cyclic adenosine monophosphate (cAMP), independently of FXR activation [40, 41].

TGR5 couples mainly with G(s) protein in several tissues [42–46]. However, paradoxical effects were observed in subtypes of cholangiocytes. In ciliary cholangiocytes, TGR5 agonists diminish cAMP levels and induce the extracellular signal-regulated kinase (ERK) signaling pathway. Still, in nonciliary cholangiocytes, TGR5 activation increased the cAMP levels and inhibited the ERK pathway, subsequently activating proliferation [42, 47]. Also, TGR5 activation has been associated with the induction of other signaling pathways, such as AKT/mTOR and NF-*κ*B [48–50].

The metabolic effects are associated with TGR5 activation. In the gastrointestinal tract, TGR5 activation induces the expression of glucagon-like peptide-1, mediating glucose homeostasis and the BA prokinetic effect [51, 52]. Besides, TGR5 activation increases the energy expenditure in brown adipose tissue by a mechanism dependent on type 2 iodothyronine deiodinase [43, 53].

Recently, our group demonstrated that DCA and CA, in a TGR5-dependent manner, induced sarcopenia and atrophy in skeletal muscle by incrementing the ubiquitin-proteasome system (UPS) and oxidative stress [44]. Also, the absence of the TGR5 receptor prevents the sarcopenia induced by cholestatic chronic liver disease, protecting the muscle from loss of mass and strength [54]. These results contradict a report indicating that TGR5 enhances muscle differentiation in the C2C12 myoblast and induces hypertrophy in mice [45]. These studies differ in the knock-out mice model and the used BA, suggesting that more analyses are necessary to understand the effect of BA in skeletal muscle and the importance of conjugation- and hydrophobicity-specific effect.

### 3.3. Other Bile Acid Receptors

The sphingosine-1-phosphate receptor 2 (S1PR2) senses the phosphorylated sphingosine and mediates mainly cell proliferation and differentiation. This membrane receptor has a high affinity to conjugated BA [55, 56]. S1PR2 activation induces the phosphorylation of ERK1/2 and AKT and reduces the BA-induced apoptosis in hepatocytes by preventing intracellular calcium oscillations [56, 57]. S1PR2 also activates the NF-*κ*B pathway through EGFR/ERK1/2/AKT, inducing a proinflammatory response [58, 59]. Besides, the absence of S1PR2 favors the development of fatty liver during a high-fat diet in mice through the sphingosine kinase 2 [60]. These antecedents suggest that BA may modulate lipid metabolism in the liver through S1PR2.

The pregnane X receptor (PXR) and constitutive androstane receptor (CAR) are intracellular sensors that mediate the detoxification process of xenobiotics [61, 62]. These receptors can bind BA and modulate the expression of genes involved in BA metabolism [63]. In this way, BA activates PXR and CAR, increasing the expression of enzymes (e.g., CYP3A, CYP2B, and sulfotransferases) that modify BA, reducing their hydrophobicity to decrease their toxicity. Besides, PXR and CAR generate diverse isoforms of BA's efflux transporters (MRP and OATP), increasing the clearance of hydrophobic BA [61, 64–66]. Therefore, both receptors complement the function of FXR by decreasing the toxicity and increasing the excretion of BA to protect the tissues from citotoxicity.

Also, the vitamin D receptor (VDR) can sense the LCA and its metabolites, but not other BA. Furthermore, VDR induces the expression of CYP3A on the small intestine and MRP3 in the colon [67, 68]. These reports suggested that VDR is a sensor that mediates the protection of the intestinal tract from toxic LCA levels.

To summarize, membrane receptors, such as TGR5 and S1PR2, are mainly associated with BA-dependent endocrine/metabolic functions in diverse tissues, unlike nuclear receptors directly related to BA homeostasis.

## 4. Bile Acid Cytotoxicity

The lipophilicity of BA is directly proportional to their cytotoxic effect due to their potential to solubilize and disrupt cell membranes. Cellular swelling, apoptosis, alterations in membrane integrity, and release of several cellular components are characteristic of BA-induced toxicity [9, 74–76]. In addition, due to the general structure, BA could induce lipid peroxidation and alterations in the lipid composition of membranes [77, 78].

**Figure 1:**
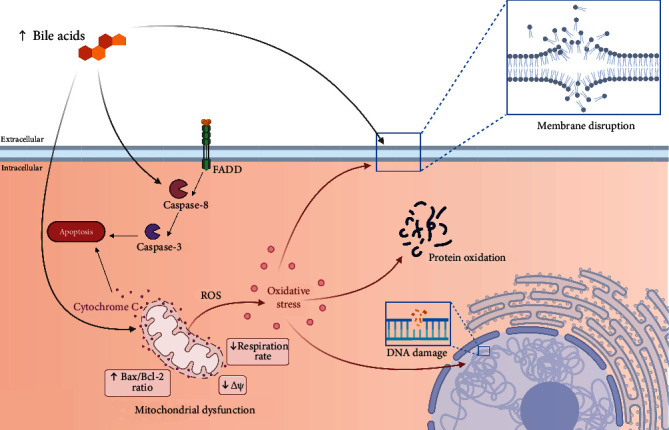
Cytotoxicity mechanisms induced by bile acids (BA). BA can induce membrane disruption by an alteration of stability and composition due to their steroid structure. Moreover, BA activate the caspase pathway in a Fas receptor-dependent mechanism (FADD), triggering cellular apoptosis. In addition, BA affect the mitochondrial function by (1) decreasing the rate of respiration, (2) diminishing the membrane potential, (3) increasing the permeability transition pore facilitating the translocation of cytochrome C and contributing to apoptosis, and (4) inducing reactive oxygen species (ROS) generation. The increased ROS levels lead to cellular oxidative stress capable of inducing DNA damage, protein oxidation, and lipid peroxidation, contributing to cellular membrane damage. All these mechanisms impair cellular viability.

In addition to membrane alterations, hydrophobic BA induce a proinflammatory response in hepatocytes by increasing membrane adhesion molecules and chemokines [79, 80]. Also, CDA and its conjugated derivatives can activate the caspase pathway in a Fas receptor-dependent mechanism [81, 82]. These antecedents indicate that BA can induce a proinflammatory response and facilitate cell death (Figure 1).

The mitochondrial function is severely affected by elevated BA levels [9, 83]. Lipophilic BA decrease the state 3 respiration and the membrane potential in mitochondria from the liver and the heart [74, 77, 84]. BA also induce the permeability transition pore and favor the release of cytochrome C into the cytosol, associated with the enhanced expression and translocation of Bax to mitochondria together with the decreased Bcl-2 expression [83, 85–88]. Furthermore, most hydrophobic BA increase mitochondrial hydroperoxide and the accumulation of compounds derived from lipid peroxidation [89]. Nevertheless, a recent report suggests that mitochondrial toxicity does not precede cytotoxicity. Other mechanisms such as lipid membrane disruption or ROS generation explain BA-dependent cytotoxicity [9]. These antecedents suggest that mitochondria are a primary target affected by BA and can be a source of oxidative stress through alterations in the electron-transport chain, favoring the cytotoxic effect (Figure 1).

It has been widely described that increased levels of lipophilic BA can induce apoptosis in diverse cell lines and tissues. However, not all BA are associated with cell damage [69, 74, 76, 90]. Particularly UDCA, the more hydrophilic BA, prevents hepatic damage by inhibiting the JNK signaling pathway and controlling the location of proapoptotic protein Bax at the mitochondrial membrane [75, 91]. Moreover, UDCA prevents the apoptosis induced by other molecules such as ethanol, TGF-*β*1, or Fas ligand, avoiding mitochondrial dysfunction and releasing cytochrome C [74, 92]. Nevertheless, coincubation of UDCA and CDCA shifts apoptosis to necrosis as the predominant cell death route in cultured human hepatocytes [83]. Similarly, the taurine-conjugated UDCA reduces the DNA fragmentation and mitochondrial dysfunction induced by ischemia in rat brains and inhibits mitochondrial efflux of cytochrome C through PI3K signaling pathway activation in rat cortical neurons [93, 94]. Also, tauro-UDCA reduces apoptosis by preventing the increase of caspase-12/Bax and the endoplasmic reticulum stress via AKT activation in mice with brain injury [95, 96].

In summary, BA can alter membranes, affecting cell structures, such as membrane and mitochondria. Besides, BA induce oxidative stress and proinflammatory response and also activate cell death pathways (Figure 1). All these mechanisms are closely associated with the structural properties of BA and have been used to explain their cytotoxicity.

## 5. Redox-Dependent Mechanisms Participate in Damage Induced by Bile Acids

The intracellular milieu is in a constant equilibrium between production and degradation of reactive oxygen, nitrogen, iron, copper, and sulfur species, generally named ROS [97]. A balanced ROS production is fundamental to normal cell function [98, 99]. ROS can be divided into radical (superoxide anion or hydroxyl radical) and nonradical species (hydrogen peroxide or hypochlorous acid, among others). ROS can be generated through enzymatic or nonenzymatic reactions [100, 101]. The intracellular oxidant species can be counterbalanced by systems that neutralize the electrophilic properties of ROS. These systems include catalase, glutathione-S-transferase, superoxide dismutase (SOD), and nonenzymatic molecules such as glutathione, thioredoxin, or vitamin E [102, 103].

Oxidative stress is established by a disturbance between ROS and antioxidants that results in excessive oxidant milieu, leading to cellular injury [97]. Oxidative stress damages cell structures by modifying proteins, lipids, nucleotides, and membranes, affecting their functions and limiting cell viability [102, 104]. To characterize and quantify oxidative stress, the ROS levels and antioxidant activity are typically determined. In addition, other parameters are end products of the oxidative modification such as lipid peroxidation (malondialdehyde (MDA), thiobarbituric acid-reactive substances (TBARS), 4-hydroxy-2-nonenal (4-HNE) or F2-isoprostanes), protein oxidation (carbonylated proteins), or even DNA oxidation (8-hydroxy-2′-deoxyguanosine (8-OHdG)) [103].

**Figure 2:**
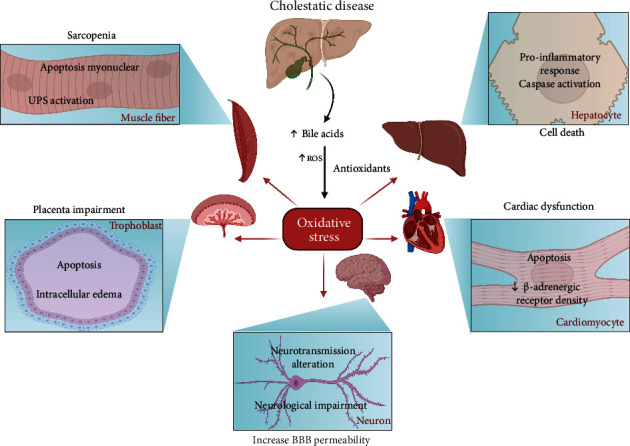
The effect of bile acid-induced oxidative stress in different tissues during a cholestatic disease. Cholestatic conditions provoke elevated serum levels of BA. Consequently, there is an imbalance between reactive oxygen species (ROS) and antioxidant systems, leading to oxidative stress. In skeletal muscle, sarcopenia is caused due to ubiquitin-proteasome system (UPS) activation and myonuclear apoptosis in fibers. In hepatic tissue, oxidative stress mediated a proinflammatory induction and caspase activation in hepatocytes. Besides, oxidative stress induces apoptosis and intracellular edema in the trophoblast during pregnancy, causing impairment in the placenta. Elevated BA levels increase the blood-brain barrier (BBB) permeability and correlated with neurological impairment and altered neurotransmission. Finally, oxidative stress induces cardiac dysfunction through apoptosis and a reduction in *β*-adrenergic receptor density in cardiomyocytes.

Below, we will detail the main effects of oxidative stress in the tissues most affected by cholestatic disorders (Figure 2).

### 5.1. Liver

Hepatocytes are highly affected by elevated BA levels [80]. Experiments in hepatocytes showed that lipophilic BA (CDCA, DCA, and CA) increase cellular hydroperoxide, superoxide anion, and TBARS production [74, 75, 89]. Also, the taurine conjugates of CDCA and CA increase the MDA levels and correlate with a decline in hepatocyte viability. This cellular toxicity was prevented by different antioxidant mechanisms [105]. These antecedents suggest that BA-induced oxidative stress affects hepatocyte viability.

FXR regulates BA homeostasis through diverse mechanisms, explaining the predominant role on cholestasis etiology [8, 26, 106]. In this line, the absence or inhibition of FXR results in a high BA concentration in serum and promotes hepatic injury [3, 69]. The *Fxr*-null mice showed an increased hepatic BA concentration causing an elevation of oxidative markers such as 8-OHdG, hydroperoxide, and TBARS. Besides, these mice also increased protective Nrf2 signaling in hepatic tissue, probably to counterbalance the cellular damage [33].

Other reports using a rat model fed with a BA-supplemented diet or a bile duct ligation model showed swollen mitochondrial and impaired cellular respiration, both associated with elevated ROS production [74, 75]. Together, these results suggest that high serum concentrations of BA induce hepatic oxidative stress.

### 5.2. Skeletal Muscle

Extrahepatic dysfunctions characterize cholestatic hepatic diseases. Among them are weakness and skeletal muscle wasting. This complex syndrome is named sarcopenia. Among the features of sarcopenia is the decreased cross-sectional area of muscle fibers due to several molecular mechanisms such as diminished protein synthesis, high protein degradation, mitochondrial dysfunction, dysregulated autophagy, and oxidative stress [104, 107, 108].

Our laboratory described the induction of sarcopenia in a mice model of cholestatic liver disease characterized by TGR5-dependent mechanisms: (1) oxidative stress, presenting elevated ROS, carbonylated proteins, and 4-HNE in skeletal muscles; (2) increased myonuclear apoptosis, with induction of the caspase pathway and increased Bax/Bcl-2 ratio; and (3) induction of protein catabolism through UPS [54, 107, 109]. Interestingly, ROS is directly associated with the UPS induction and mitochondrial alterations that might induce apoptosis [108, 110–112]. In addition, the use of an antioxidant treatment (N-acetyl cysteine) prevents muscle damage and diminishes the apoptotic effect [109].

Moreover, recently it has been described that CA and DCA resemble the skeletal muscle atrophy induced by cholestatic liver disease, UPS induction, and oxidative stress. Also, the absence of TGR5 in muscle fibers abolished all harmful effects caused by these BA [44]. Thus, all these antecedents firmly suggest that elevated BA in cholestatic disorders induce oxidative stress through the TGR5 receptor, activating several intracellular events that cause sarcopenia.

A recent study has shown a relationship between muscle-BA-gut microbiota. Results indicate that the alteration of gut microbiota induced sarcopenia. This muscle dysfunction was associated with an altered profile of BA that reaches the portal blood circulation. This change induces the inhibition of ileal FXR signaling with the consequent decrease in serum levels of FGF15, an enterokine related to muscle wasting [113]. Considering the antecedents related to muscular TGR5, BA, and sarcopenia [44, 54], it is impossible to discard this receptor's participation in the muscle dysfunction associated with alteration in the microbiota-BA axis.

### 5.3. Central Nervous System

Oxidative stress is crucial in hepatic encephalopathy, and altered BA levels (elevated, changes on conjugated/unconjugated and primary/secondary ratio) could be associated with neurological decline [104, 114–116]. It was described that BA, via Rac1 activity, increase the blood-brain barrier permeability, facilitating the neurological changes associated with cholestatic diseases [116]. Also, patients with Alzheimer's disease present with increased secondary and conjugated BA levels that correlate with the disease's advanced stages [117]. Furthermore, CA, DCA, and CDCA modulate the respiratory-related rhythmic discharge activity in an FXR-dependent manner, which interferes with NMDA or GABA neurotransmission, suggesting that BA affects the brain's normal function [118, 119].

The FXR and TGR5 receptors have been associated with neurological damage. Downregulation of FXR in the frontal cortex replicated the neuroprotective effect of reducing BA levels in mice with acute liver failure, suggesting that FXR signaling mediates the neurological decline in this model [114]. Additionally, the absence of FXR correlates with reduced brain infarct volume, prevents neuronal apoptosis by an anti-inflammatory response, and reduces calcium influx after oxygen-glucose deprivation in a cerebral ischemia mice model [120]. Further, the TGR5 receptor in astrocytes responds to neurosteroids, molecules with structural similarities with BA, elevating the intracellular calcium and ROS [10]. Those results confirm that the BA receptors could be relevant in generating oxidative stress and neurological impairment.

Nevertheless, tauro-UDCA prevents lipopolysaccharide depressive-like mice model, an effect that correlated with neuroinflammatory protection and decreased MDA/nitrite levels in the hippocampus and prefrontal cortex [121]. Similarly, the hydrophobic CA induced anti-inflammatory properties and reduced oxidative stress (decreasing MDA, NO, Il-1*β*, and TNF-*α*) in an integrative functional unit composed of neurons and neural supporting cells known as the neurovascular unit [12]. The prevention of oxidative stress and neuroprotective effect might be related to the TGR5 receptor. Its activation with a semisynthetic agonist decreased oxidative stress and neuronal apoptosis and downregulated the NF-*κ*B pathway in mice with brain injury [37, 71]. Those results suggest that BA might have different roles in the oxidative stress induction in CNS in noncholestatic conditions.

Considering oxidative stress with neurological pathologies and the conflicting description of BA on the oxidative stress in CNS, it is crucial to perform more mechanistic analysis to understand BA's role. Also, understanding the BA-CNS relation raises the possibility of proposing novel pharmacological strategies, including BA receptor modulation, for neurological disorders and neurodegenerative pathologies.

### 5.4. Heart

During cholestatic diseases, serum BA elevation is associated with direct toxic effects on the heart and the impairment of myocardial function [35, 90, 122]. In addition, CDCA induces apoptosis in neonatal rat ventricular myocytes due to the loss of mitochondrial membrane potential and cytochrome C release, as well as consequent caspase-pathway activation. The bile duct ligation model resembles the cardiac proapoptotic response and shows an impairment contractibility [82, 87].

Also, CA decreases the heart rate and myocardial contraction and increases the markers of cardiac injury concomitantly with decreased *β*-adrenergic receptor density. These characteristics resemble the cardiac alteration in the FXR knock-out model and cholestatic liver disease [35, 123]. Interestingly, FXR inhibition suppresses cardiac apoptosis. Additionally, FXR inhibition in an ischemia-reperfusion model reduces cardiotoxicity and decreases myocardial infarct size improving cardiac function [87]. However, a contradictory report showed that FXR agonists activate the Nrf2 signaling (decreasing ROS, MDA, and 8-OHdG through elevated catalase, glutathione-S-transferase, and SOD), preventing cardiomyopathy in a diabetic mice model [124]. Those results suggest that BA directly and via oxidative stress could mediate the cardiotoxicity. However, the protective effect of FXR must be analyzed deeply.

Furthermore, BA also activate TGR5 in ventricular myocyte cell culture [46]. Selective TGR5 agonist (INT-777) prevents NF-*κ*B activation and decreases the ROS level induced by high glucose treatment in primary cardiomyocytes [125]. Moreover, LCA prevents high glucose-induced hypertrophy in the cardiac myoblast cell line, and TGR5 upregulation alleviates the oxidative stress and inflammatory process through activating the AKT pathway in the cardiac myoblast cell line [126–128]. Also, the TGR5-dependent protective effect induced by BA *in vivo* was described with the administration of DCA in a cardiac injury mice model, improving cardiac remodeling and inhibiting the proinflammatory response [128]. These results suggest that TGR5 has a protective role in myocardial tissue associated with diminishing oxidative stress.

In general, BA can exert opposing effects on the myocardial tissue depending on the mediated receptor involved. All those results indicate that BA can be one of the responsible causes of cardiac impairment by several mechanisms in cholestasis. However, TGR5 showed a promissory pharmacological target.

### 5.5. Placenta

Intrahepatic cholestasis in pregnancy increases the risk of adverse outcomes, even causing intrauterine death [69]. The placenta has a protective role to the fetus from molecules of different structural nature. During pregnancy, the increased serum BA impaired the protective function of the placenta and enhanced the toxicity to the fetus [86, 129]. Studies using trophoblast cell lines and diverse gestational cholestatic animal models showed edema and apoptosis in the placenta, attenuating with FXR agonist or UDCA treatments [69, 86, 130, 131]. Also, UDCA has been successfully proved in intrahepatic cholestasis in pregnancy patients without interfering with the placental hormone production and with no-fetal side effects. However, it does not enhance the perinatal death ratio, BA concentration, and itch score [132–134].

The elevated BA levels are associated with the increase of oxidative stress markers (MDA and carbonylation proteins) in the placenta, as well as the decrease of antioxidant gene expression and activity of catalase, glutathione-S-transferase, SOD, peroxiredoxin (PRDX), among others [69, 86]. Also, increased MDA levels and diminished expression of PRDX1 and PRDX3 were reported in the placenta from intrahepatic cholestasis of pregnant human patients [69]. These results suggest that oxidative stress induced by BA mediates the placenta's impairment and contributes to the affectation in the mother and fetus.

## 6. Clinical Perspective and Conclusions

BA are amphiphilic molecules mainly characterized by their ability to form micelles, and they are associated with nutrient absorption at the intestinal level. However, BA have endocrine functions that regulate metabolic activity and cellular energy through facilitating lipid- and carbohydrate metabolism. Several receptors are associated with BA-dependent actions, such as FXR, TGR5, S1PR2, PXR, CAR, and VDR. Indeed, FXR and TGR5 have been widely studied to understand BA effects and propose novel therapeutics for cholestatic disorders.

The FXR receptor has a central role in BA physiology and carbohydrate and lipid homeostasis. Most cholestatic disorders are characterized by BA transport impairments associated with FXR malfunction, making this receptor an attractive molecular target to treat cholestasis [1, 32, 135]. Also, it has been reported that UDCA decreased FXR activation and increased triglycerides in obese patients [136]. This evidence enhances the interest in developing FXR activators. Interestingly, some non-BA molecules that can activate FXR have been tested in preclinical studies [137–140].

Conversely, the TGR5 receptor has a pivotal role in cell differentiation in some cell lines, and its activation is also associated with diverse signaling pathways [42, 48]. Also, TGR5 activation was associated with upregulation of type 2 iodothyronine deiodinase, increased production of glucagon-like peptide-1, and even intestinal motility [43, 52, 141, 142]. Since the TGR5 functions are related to metabolism and there exists the need for treating metabolic diseases such as diabetes or obesity, there is an increased interest in finding novel TGR5 agonists [5, 143–145].

Due to the relevance on metabolism and gastrointestinal physiology, BA receptors have been studied as a pharmacological target to treat some diseases. Indeed, some BA such as UDCA, CA, DCA, and CDCA, have been clinically approved by the U.S. Food and Drug Administration (FDA) to treat some pathological conditions. For example, they can dissolve and prevent gallstone (UDCA), primary biliary cirrhosis (UDCA, CDCA, and obeticholic acid), BA synthesis disorders (CA), and more recently, they have been used to improve the appearance of submental fat (DCA) [146, 147].

Despite these antecedents, BA or modified BA are still under clinical research to approve their therapeutic indication. The potential use of BA as a treatment for pathologies has been established in phase I clinical trials. Thus, TUDCA and CDCA administration improves insulin sensitivity through increased glucagon-like peptide-1 secretion in patients with obesity and diabetes. Besides, UDCA administration induces hepatic-protective properties after radiation [148–151]. A combination of taurine-UDCA and phenylbutyrate demonstrated prevention of functional decline and prolonged survival in patients with amyotrophic lateral sclerosis [152, 153]. Together, these clinical studies suggest that BA have promising effects on nongallbladder pathologies. Nevertheless, more advanced clinical trials are needed to demonstrate that BA can be used in these conditions and the eventual relationship with oxidative stress.

Treatment with BA generates unwanted side effects such as diarrhea/excessive flatus and pruritus. Interestingly, modified BA and non-BA FXR agonists are helpful to prevent those adverse effects. The diarrhea is associated with alteration in secretion and motility in the colon, and activation of FXR by obeticholic acid or tropifexor (non-BA FXR agonist) increases the feedback inhibition via fibroblast growth factor 19, improving diarrhea scores [154, 155].

Although several clinical trials with BA failed to improve nonalcoholic steatohepatitis, the obeticholic acid improved hepatic histology, decreased fibrosis, and increased insulin sensitivity [156–158]. Also, obeticholic acid reduced serum alkaline phosphatase level in patients with primary biliary cholangitis [159]. However, similar to other BA, obeticholic acid developed pruritus in patients in different clinical trials [156, 159, 160]. The beneficial effect without this secondary effect was obtained by using 24-*nor*-UDCA in patients with primary sclerosing cholangitis or nonalcoholic fatty liver disease [157, 161]. These reports suggest that modified BA could be the better option for future treatments. However, long-term studies with modified BA are needed to analyze the relevance of side-effect prevention, as well as the relation with oxidative stress.

Furthermore, the agonism of TGR5 or FXR could be inappropriate in other tissues, mainly in the skeletal muscle, heart, and gallbladder, presenting some adverse effects [162–164]. Recently, it has been reported that obeticholic acid may increase the gallstone risk by a mechanism dependent on FXR activation and FGF19 participation [165]. However, it is essential considering the severe side effects, mainly with long-term and high-dose BA treatments, as UDCA was associated with increased risks of developing colorectal neoplasia in primary sclerosing cholangitis, and its withdrawal deteriorates liver serum markers and increases pruritus [166–168].

More recently, there is an interest in developing FXR/TGR5 dual agonists due to an eventual synergistic effect [169]. Some beneficial effects have been reported in preclinical studies of kidney disease and liver steatosis through anti-inflammatory mechanisms [170–173]. However, there are only initial reports, and additional research is necessary to establish the relevance of this dual strategy.

The cellular alterations induced by elevated BA levels include membrane damage, proinflammatory response, mitochondrial dysfunction, and cell death by apoptosis or necrosis. All of these effects are directly or indirectly related to redox-dependent mechanisms. Interestingly, in hepatic tissues, oxidative stress and cellular damage are closely associated with FXR signaling. Meanwhile, in skeletal muscle, BA-induced injury is a TGR5-dependent process. In all mentioned tissues in this review (hepatic, skeletal muscle, CNS, heart, and placenta), oxidative stress has a significant role in apoptosis. However, an evidence gap indicates that additional research must be performed to understand and establish the complex signaling involved in the potential harm of the BA-oxidative stress axis and the long-term effect of BA as a therapeutical option. In the same direction, BA-induced redox signaling is a central hallmark that could be considered a target for developing innovative therapeutic options to treat cholestatic diseases.

## Figures and Tables

**Table 1:** The general structure of more abundant bile acids in humans.

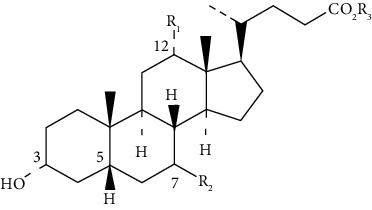			
Bile acid	R1	R2	R3
Cholic acid	OH	*α*-OH	Acid form 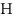 Glycine-conjugate 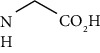 Taurine-conjugate 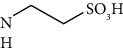
Chenodeoxycholic acid	H	*α*-OH	
Deoxycholic acid	OH	H	
Lithocholic acid	H	H	
Ursodeoxycholic acid	H	*β*-OH	

**Table 2:** Bile acid receptor distribution and ligands.

Receptor	Classification	Distribution	Main agonist	References
FXR	Nuclear receptor	Liver and intestine Minor expression in the heart, kidneys, CNS, white adipose tissue, adrenal gland, pancreas, and placenta	CDCA > LCA = DCA > A 7*α*‐OH ≫ 7‐keto ≫ 7*β*‐OH	[4, 20, 69]
PXR	Nuclear receptor	Liver and intestine Kidney, stomach, and CNS	LCA ≈ CDCA ≈ CDA	[61, 64]
CAR	Nuclear receptor	Liver, kidney, CNS, and adrenal gland	LCA	[65]
VDR	Nuclear receptor	Small intestine, colon, skin, heart, and kidney	LCA and metabolite	[67, 70]
TGR5	G-protein-coupled receptor	Heart, skeletal muscle, lung, spleen, kidney, liver, CNS, enteric nervous system, gastrointestinal tract, placenta, and adipocytes	LCA > DCA > UDCA > CDCA > CA	[10, 40, 46, 47, 71]
S1PR2	G-protein-coupled receptor	Liver, small intestine, CNS, and enteric nervous system	Conjugated DCA ≈ conjugated CA	[72, 73]

## Data Availability

Data is available on request.

## Abbreviations

4-HNE: 4-Hydroxy-2-nonenal
8-OHdG: 8-Hydroxy-2′-deoxyguanosine
AKT: Protein kinase B
BA: Bile acids
Bax: Bcl-2-associated X
Bcl-2: B-cell lymphoma 2
BSEP: Bile salt export pump
CA: Cholic acid
cAMP: Cyclic adenosine monophosphate
CAR: Constitutive androstane receptor
CDCA: Chenodeoxycholic acid
CNS: Central nervous system
CYP: Cytochrome
DCA: Deoxycholic acid
DNA: Deoxyribonucleic acid
EGFR: Epidermal growth factor receptor
ERK: Extracellular signal-regulated kinases
FXR: Farnesoid X receptor
LCA: Lithocholic acid
LPS: Lipopolysaccharide
MDA: Malondialdehyde
MRP: Multidrug resistance proteins
mTOR: Mammalian target of rapamycin
NF-*κ*B: Nuclear factor kappa beta
OATP: Organic anion transporting polypeptide
OST: Organic solute transporter
PRDX: Peroxiredoxin
PXR: Pregnane X receptor
ROS: Reactive oxygen species
S1PR2: Sphingosine-1-phosphate receptor2
SLC: Solute carrier
SOD: Superoxide dismutase
TBARS: Thiobarbituric acid-reactive substances
TGF-*β*1: Transforming growth factor-beta type 1
TGR5: Takeda-G-protein-receptor-5
UDCA: Ursodeoxycholic acid
UPS: Ubiquitin-proteasome system
VDR: Vitamin D receptor.
